# Navigating the complexities of ferroptosis in pancreatic ductal adenocarcinoma: roles, mechanisms and potential applications

**DOI:** 10.1038/s41420-026-02987-2

**Published:** 2026-02-26

**Authors:** Yurao Xiao, Wenjia Wang, Guihua Wang, Yuhui Liu, Jun Gong

**Affiliations:** 1 Division of Hepato-Pancreato-Biliary Surgery, Tongji Hospital, Tongji Medical College, Huazhong University of Science and Technology, Wuhan, Hubei China; 2 Hubei Key Laboratory of Hepato-Pancreato-Biliary Diseases, Wuhan, Hubei China; 3 GI Cancer Research Institute, Tongji Hospital, Tongji Medical College, Huazhong University of Science and Technology, Wuhan, Hubei China; 4 Division of Child Healthcare, Department of Paediatrics, Tongji Hospital, Tongji Medical College, Huazhong University of Science and Technology, Wuhan, Hubei China

**Keywords:** Cancer metabolism, Cell death, Biomarkers, Cancer microenvironment

## Abstract

Pancreatic ductal adenocarcinoma (PDAC) is a lethal malignancy with limited therapeutic options and a profoundly immunosuppressive tumor microenvironment (TME). Ferroptosis, a novel form of regulated cell death driven by iron-dependent lipid peroxidation, has emerged as a promising therapeutic avenue by targeting metabolic vulnerabilities in cancer cells. Notably, key ferroptotic pathways in PDAC involve iron accumulation, lipid peroxidation, and oxidative stress. Major defense systems include the System Xc⁻/GSH/GPX4, NAD (P)H-FSP1-CoQH_2_/VKH_2_, DHODH-CoQH_2_, and GCH1-BH4 pathways. Ferroptosis exhibits dual roles in PDAC, demonstrating both tumor-suppressive and oncogenic effects within TME. Ferroptosis-related biomarkers show promise for PDAC diagnosis and prognosis. Novel therapeutic strategies combining ferroptosis inducers with conventional treatments and nanoparticle-based delivery systems have shown encouraging results in preclinical studies. While ferroptosis-based therapies offer potential for PDAC treatment, challenges remain in translating these approaches to clinical practice. Therefore, this review provides a comprehensive synthesis of the mechanistic insights, therapeutic potential, and associated challenges of targeting ferroptosis in PDAC. It is necessary to identify specific biomarkers, mitigate side effects, and elucidate the complex interactions between ferroptosis and TME. Integrating ferroptosis modulation with existing therapies may lead to more effective, personalized treatment strategies for PDAC.

## Facts

- PDAC stands as one of the most lethal solid malignancies worldwide, characterized by a highly aggressive phenotype, a profoundly immunosuppressive TME, and notorious resistance to conventional and emerging immunotherapies.
- The core mechanisms of ferroptosis converge on the unique metabolic vulnerabilities of PDAC cells.
- The clinical application of ferroptosis-based biomarkers and therapies shows great prospect in PDAC diagnosis and treatment.

## Introduction

Pancreatic cancer, predominantly manifested as PDAC, stands as one of the most lethal solid malignancies worldwide. Its dismal prognosis, with a 5-year survival rate below 10%, is largely attributable to late diagnosis, early metastatic dissemination, and profound resistance to conventional chemotherapy and radiotherapy [1]. The aggressive biology of PDAC is further compounded by a characteristically dense, desmoplastic stroma that fosters an immunosuppressive TME, contributing to immune evasion and treatment failure [2].

In 2012, Dixon et al. identified ferroptosis as a distinct form of regulated cell death, mechanistically characterized by the iron-driven accumulation of lethal lipid peroxides. This process is defined by the peroxidation of polyunsaturated fatty acid-containing phospholipids (PUFA-PLs), leading to membrane damage and cell death [3, 4]. The execution of ferroptosis is counterbalanced by a sophisticated array of cellular defense systems, such as the system Xc⁻/glutathione (GSH)/GPX4 axis and the NAD (P)H-FSP1-CoQH₂ pathway, which work in concert to neutralize lipid peroxides and maintain redox homeostasis [5–7].

Compared with normal cells, cancer cells have been shown to require higher levels of iron, which renders them more susceptible to ferroptosis [8–10]. Thus, targeting ferroptosis defense and execution mechanisms represents a promising strategy for the treatment of refractory cancers, including PDAC. The regulation of ferroptosis to inhibit PDAC cell growth has become a significant focus of PDAC therapy. This article reviews the current understanding of ferroptosis mechanisms and explores their therapeutic applications in PDAC treatment.

## Characteristics of ferroptosis

Ferroptosis is distinguished from other forms of cell death by its unique morphological, biochemical and genetic characteristics (Table 1). Morphologically, ferroptotic cells exhibit plasma membrane bubbling and shrunken mitochondria. This profile clearly differs from apoptosis, which is characterized by cell shrinkage, nuclear fragmentation, and apoptotic body formation, and from pyroptosis, which features rapid cell swelling and extensive plasma membrane pore formation [11, 12]. Biochemically, ferroptosis is driven by the accumulation of intracellular free iron and the generation of reactive oxygen species (ROS), particularly lipid ROS. These deleterious molecules are primarily generated through Fenton and Fenton-like reactions between hydrogen peroxide and ferrous iron, culminating in excessive lipid peroxidation [3, 13]. This accumulation of lipid peroxides is lethal to the cell and is a key feature that differentiates ferroptosis from other cell death pathways. Importantly, ferroptosis is caspase independent and occurs when the antioxidant defense system, primarily the system Xc-/GSH/GPX4 axis, is compromised. Consequently, the compromise of these ferroptosis defense systems results in the lethal accumulation of lipid peroxides, which directly executes ferroptotic cell death. Genetically, ferroptosis is regulated by a series of key regulatory genes. For example, ACSL4 facilitates the incorporation of PUFAs into phospholipids, increasing their susceptibility to peroxidation [14]. SLC7A11 plays a crucial role in cysteine uptake, which is necessary for GSH synthesis. GPX4 utilizes GSH to convert lipid peroxides into nontoxic lipid alcohols, thereby preventing lipid peroxidation. Additionally, FSP1 catalyzes the reduction of coenzyme Q10 (CoQ_10_) or vitamin K into their reduced forms, CoQH_2_ or VKH_2_, which function as antioxidants to suppress ferroptosis [5, 15]. The balance between ferroptosis execution and defense mechanisms ultimately determines whether a cell undergoes ferroptosis.

**Table 1:** Biochemical features, key regulatory genes, morphological features, immune features, induction mechanisms and biological effects of cell death.

	Ferroptosis	Necroptosis	Apoptosis	Autophagy	Pyroptosis	Alkaliptosis
Biochemical features	Accumulation of intracellular free iron, generation of ROS and lipid peroxides, caspase-independent	RIPK1/RIPK3-mediated phosphorylation of MLKL, cytosolic necrosome formation, caspase-independent	Activation of caspases, DNA fragmentation, phosphatidylserine exposure	DNA fragmentation	Activation of CASP1, CASP3, and GSDM, caspase-dependent GSDMD or GSDME cleavage, IL-1β release	Caspase-independent intracellular alkalinization
Key regulatory genes	GPX4, SLC7A11, FSP1, NFE2L2, ACSL4, NCOA4, TFRC, TP53	RIPK1, RIPK3, MLKL, LUBAC and AURKA	CASP3, CASP9, CASP8, TP53, BAX, BAK1, BCL-2	BECN1, AMPK, mTOR	CASP1, CASP11, GSDMD, GPX4, ESCRT-II, PKA	IKBKB (IKKB), NF-KB, CA9 and SLC9A7
Morphological features	Cell swelling, shrunken mitochondria with reduced or absent cristae, increased membrane density, the formation of membrane pores, plasma membrane bubbling	Cell swelling, pore formation on cell membrane, moderate chromatin condensation	Apoptotic body formation, cell shrinkage, membrane blebbing, chromatin condensation,	Formation of double membraned autolysosomes	Cell swelling, pore formation on cell membrane, plasma membrane bubbling, moderate chromatin condensation	Necrosis-like morphology
Immune features	ICD	ICD	TCD or ICD	ICD	ICD	ICD
Inducing mechanisms	Iron metabolism disorder, accumulation of lipid peroxides, antioxidant system imbalance	Multiple innate immune signaling pathways such as those initiated by the stimulation of RIG-I-like receptors, TLRs and death receptors	Genotoxic stress, the binding of ligands to cell surface death receptors	Nutrient deprivation, hypoxia and oxidative stress induced autophagosome formation	Pathogen invasion	the lethal rise in intracellular pH
Physiological and pathological implications	Anticancer therapies, neurodegenerative and iron overload disorders, inflammatory diseases	Eliminate pathogen-infected cells and/or damaged cells during certain degenerative or inflammatory disorders	Remove damaged cells	Maintain intracellular homeostasis, suppress tumor	Defend the organism against pathogens	Suppress tumor
DAMPs	HMGB1	DNA and IL-6	Ecto-CRT, HMGB-1, ATP	HMGB-1	HMGB1, ATP, IL-1β and IL-18	HMGB1
Refs.	[12, 170, 171]	[172, 173]	[174–176]	[177–179]	[174, 176, 180–182]	[183, 184]

*ICD* immunogenic cell death, *TCD* tolerogenic cell death, *GPX4* glutathione peroxidase 4, *SLC7A11* solute carrier family 7 member 11, *HMGB1* high mobility group protein B1, *RIPK1* receptor-interacting serine/threonine kinase 1, *RIPK3* receptor-interacting serine/threonine kinase 3, *MLKL* mixed lineage kinase domain-like pseudokinase, *AURKA* aurora kinase, *ESCRT* endosomal sorting complex required for transport, *RIG-I* retinoic acid-inducible gene I, *TLRs* Toll-like receptors, *IL-6* interleukin-6, *p53* tumor protein p53, *Bax* BCL2-associated X protein, *Bak* Bcl-2 homologous antagonist/killer, *Bcl-2* B-cell lymphoma 2, *Ecto-CRT* extracellular calreticulin, *HMGB1* high mobility group box 1 protein, *GSDMD* gasdermin D, *GSDME* gasdermin E, *CASP1* caspase-1, *CASP11* caspase-11, *PKA* protein kinase A, *IL-1β* interleukin-1 beta, *IL-18* interleukin-18, *IKBKB (IKKB)* inhibitor of nuclear factor kappa b kinase subunit beta, *NF-KB* nuclear factor kappa-light-chain-enhancer of activated B cells, *CA9* carbonic anhydrase 9, *SLC9A7* solute carrier family 9 member 7.

## Molecular mechanisms of ferroptosis in PDAC

### Iron metabolism and PDAC

Iron is crucial for multiple fundamental biological processes within cells. Iron plays a key role in oxygen transport as a component of heme in hemoglobin, serves as a cofactor for ribonucleotide reductase in DNA synthesis, and is indispensable for ATP production supporting enzymes in the citric acid cycle and the electron transport chain. To sustain normal cellular functions, intracellular iron levels must be precisely regulated. However, iron is also oxidatively active, catalyzing the production of ROS through Fenton and Fenton-like reactions, which induce oxidative stress and trigger ferroptosis [16]. Thus, maintaining iron homeostasis is crucial for preventing oxidative damage and ensuring cellular survival.

**Fig. 1:**
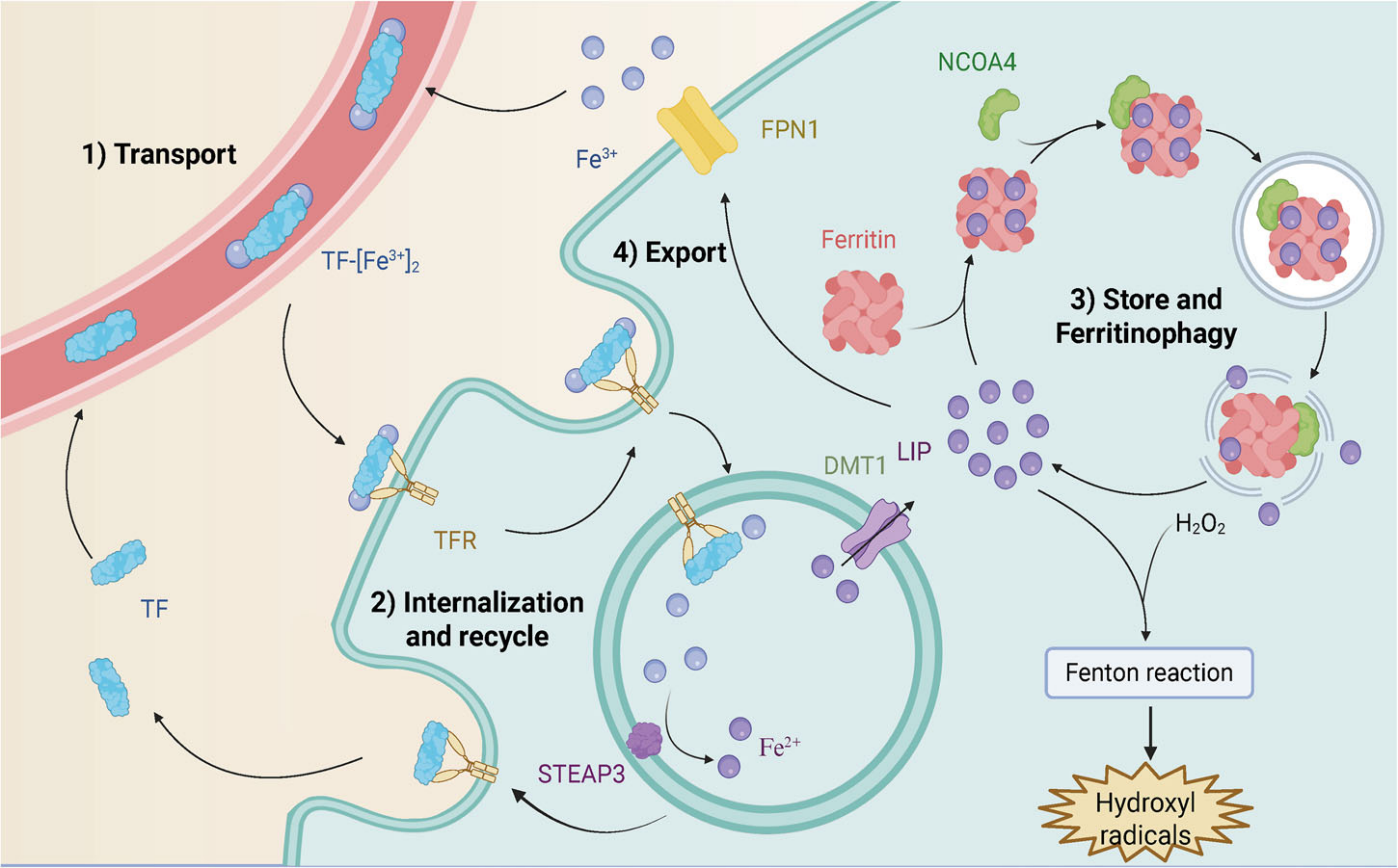
Core mechanisms of iron metabolism. 1) Transport: Fe^3+^ is transported by binding to TF. 2) Internalization and recycle: TF-Fe^3+^ binds to TFR1 at the membrane, and Fe^3+^ is internalized and reduced to Fe^2+^ by STEAP3 to enter the active iron pool, and the apotransferrin-TFR1 complex is recycled. 3) Store and ferritinophagy: excess iron is stored with ferritin and can be degraded by NCOA4 to release iron to the LIP again. 4) Export: FPN1 exports iron in the LIP from the cell to maintain iron homeostasis. TF transferrin, TFR transferrin receptor 1, FPN1 ferroportin, NCOA4 nuclear receptor coactivator 4, DMT1 divalent metal transporter 1, LIP labile iron pool, STEAP3 metalloreductase STEAP3. This figure was created with BioRender.com.

The regulation of iron homeostasis depends on various iron metabolism proteins. Dietary iron, which is absorbed in the duodenum, binds to transferrin (TF) in the form of Fe^3+^ for transport. The TF-Fe^3+^ complex then binds to transferrin receptor 1 (TFR1) at the plasma membrane and is internalized through endocytosis [17]. In the acidic environment of the endosome, ferric iron is released from TF and reduced to ferrous iron (Fe^2+^) by the ferrireductase STEAP3. The apotransferrin-TFR1 complex is then recycled back to the cell surface for further iron uptake. Moreover, ferrous iron is transported from the endosome into the cytoplasm via divalent metal transporter 1 (DMT1), entering the metabolically labile iron pool (LIP). Within this pool, the iron is available to participate in essential biological processes. Excess iron is stored in ferritin, a multimer composed of heavy and light chains, which sequesters iron to prevent its toxic accumulation [16]. Ferroportin (FPN1), an iron efflux protein, plays a critical role in maintaining cellular iron levels by exporting excess iron from the cell. Ferritinophagy is the process of the autophagic degradation of ferritin and is mediated by the nuclear receptor coactivator (NCOA4), which releases stored iron into the LIP [12]. These iron-regulating proteins play critical roles in maintaining iron homeostasis and determining cellular sensitivity to ferroptosis (Fig. 1).

Cancer cells, including PDAC cells, exhibit an increased demand for iron to support their accelerated proliferation. This heightened requirement leads to dysregulated iron homeostasis, which is characterized by altered expression of iron-regulating proteins. For example, cancer cells often upregulate proteins that increase intracellular iron levels, such as TFR1, IREB2, STEAP3, and hepcidin [18, 19]. Mitochondrial iron overload plays a significant role in promoting PDAC tumorigenesis. Excess mitochondrial iron generates ROS, which enhances glycolysis and triggers the Warburg effect, thus fueling the rapid growth of cancer cells. Moreover, mitochondrial iron activates the inflammasome AIM2, further promoting tumor development [20].

Advances in iron metabolism manipulation have yielded promising results for regulating ferroptosis in patients with PDAC. For example, the combination of dihydroartemisinin (DHA) and cisplatin has been found to increase transferrin receptor levels on the cell membrane while promoting the degradation of NCOA4, a key regulator of ferritinophagy. This degradation releases free iron from ferritin, leading to the accumulation of unstable iron, which contributes to ferroptosis induction [21]. Similarly, trametinib (a MEK inhibitor) enhances the transcription of ferritin heavy chain (FTH1) and ferritin light chain (FTL) and promotes ferritinophagy through NCOA4 while reducing ferroportin expression, thereby increasing intracellular iron levels and ultimately triggering ferroptosis in PDAC cells [22]. The stress-inducible transcription factor NUPR1 targets lipocalin-2 (LCN2), an iron-transporting protein that, along with FPN1, releases Fe^2+^ into the extracellular space to suppress ferroptosis [23]. In addition, the inhibition of aspartate aminotransaminase GOT1 promotes labile iron release in PDAC through NCOA4-dependent ferritinophagy, highlighting GOT1 as a therapeutic target through the promotion of PDAC ferroptosis [24]. Additionally, the organic selenium compound DBDS disrupts mitochondrial integrity and facilitates the release of iron from mitochondria, resulting in an increase in the LIP and the induction of ferroptosis in PDAC. Notably, DBDS can be incorporated into composite nanoarchitectures (DBDS-NAs) to improve drug delivery, further potentiating its role as a ferroptosis-inducing agent in the treatment of PDAC [25].

### Lipid metabolism and PDAC

Lipids are essential components of biological membranes and are crucial in cellular structure. Lipids are also vital for energy storage and metabolism and serve as important signaling molecules in various cellular activities. Lipid metabolism is critical for maintaining intracellular homeostasis. In cancer cells, nutrient availability fluctuates during tumor progression, and lipid metabolism significantly contributes to rapid proliferation, survival, migration, invasion, and metastasis [26]. Additionally, lipids provide necessary building blocks for the rapid formation of cancer cell membranes and serve as substrates and signaling molecules for the posttranslational modification of proteins, thereby accelerating tumor progression [27].

PDAC cells exhibit distinct alterations in lipid metabolism. For example, tissue-resident pancreatic stellate cell-derived cancer-associated fibroblasts (CAFs) secrete lipids, such as lysophosphatidylcholines, which promote the absorption and synthesis of phosphatidylcholine by cancer cells [28]. In terms of lipogenesis, the phosphorylation of INSIG1 at S207 disrupts its ability to inhibit SREBP, a key regulator of adipogenesis, thereby sustaining elevated lipogenesis in tumor cells [29]. Additionally, the fatty acid oxidation (FAO) pathway is upregulated in PDAC cells, leading to increased FAO activity [30]. These cells also increase their invasive potential by storing fatty acids (FAs) in lipid droplets, which can be utilized as an energy source under lipase-driven conditions to fuel metastatic invasion. Notably, KRAS mutations reduce lipase levels, thereby increasing lipid storage and suppressing FA metabolism, which allows stored lipids to further support invasion and metastasis [31].

Ferroptosis is driven by the uncontrolled generation of PUFAs-containing lipid peroxides within the cell membrane [32]. ACSL4 catalyzes the binding of long-chain PUFAs, such as arachidonic acid (AA) and adrenic acid (AdA), to coenzyme A (CoA) [33], after which these products are re-esterified by lysophosphatidylcholine acyltransferase 3 (LPCAT3) to form phospholipids [34]. Additionally, conjugated linoleic acids, such as α-ESA, are acylated and integrated into cellular phospholipids through the action of ACSL1 [35]. During lipid peroxidation, the PUFAs that make up the membrane phospholipids lose bis-allylic hydrogen atoms, resulting in the formation of phospholipid radicals (PL·) [34]. These radicals subsequently react with molecular oxygen to generate phospholipid peroxyl radicals ((P)LOO), which further interact with PUFA phospholipids, extracting hydrogen atoms and producing phospholipid hydroperoxides ((P)LOOHs) [34, 36]. This chain reaction ultimately compromises cell membrane integrity, leading to membrane rupture and the inhibition of cell growth [37]. Under normal conditions, GPX4 utilizes GSH as a co-substrate to reduce (P)LOOH to non-toxic lipoalcohols ((P)LOH), inhibiting the occurrence of ferroptosis [38]. On the other hand, saturated fatty acids (SFAs) are converted to monounsaturated fatty acids (MUFAs) via stearoyl-CoA desaturase 1 (SCD1). MUFAs, such as oleic acid, are acylated by ACSL3 and incorporated into cellular phospholipids. MUFAs can inhibit the accumulation of ROS in the plasma membrane and reduce the binding of PUFAs to phospholipids, thereby mitigating oxidative processes and preventing ferroptosis [39].

Various drugs and proteins can modulate ferroptosis in PDAC by influencing lipid metabolism pathways. PUFAs such as linoleic acid (LA), α-linolenic acid (αLA), eicosapentaenoic acid (EPA) and AA have been shown to induce ferroptosis and inhibit the proliferation of PDAC cells [40]. Further investigations revealed varying sensitivities to PUFA-induced ferroptosis across different cancer cell lines, suggesting that the lipid metabolic profile of cells plays a significant role in determining ferroptosis sensitivity [40]. Gemcitabine induces CAFs to secrete exosomes containing miR-3173-5p, which inhibits the expression of ACSL4, thereby decreasing PUFA binding to phospholipids and enhancing resistance to ferroptosis in PDAC cells [41, 42]. FBW7, a substrate-recognition component of the SCF ubiquitin ligase complex, inhibits the transcription of SCD1 by antagonizing the transcription factor NR4A1, which subsequently decreases MUFA generation from SFAs and reverses resistance to ferroptosis. MEN1, a key driver of pancreatic neuroendocrine tumors (pNETs), suppresses SCD1 expression by inhibiting mTOR, resulting in a decrease in MUFAs such as oleic acid (OA) and ultimately promoting lipid peroxidation and ferroptosis in pNETs. Mutation of MEN1 abolishes its tumor-suppressor role by inducing ferroptosis and promoting the progression of pNET [43]. Additionally, pirin (PRI), an iron-binding nuclear protein, plays a critical role in nuclear stress during ferroptosis by downregulating ACSL4 mRNA and its product, AA, thereby inhibiting ACSL4-mediated ferroptosis in PDAC [44].

### Oxidative stress and PDAC

ROS are partially reduced or excited forms of oxygen, including superoxide (O₂−), hydrogen peroxide (H₂O₂), and hydroxyl radicals (OH·), which can rapidly interconvert with each other. For example, superoxide can be transformed into hydrogen peroxide by superoxide dismutase (SOD), which transitions from a highly reactive, short-lived form to a relatively stable state. Hydrogen peroxide can be further converted into water and molecular oxygen under the action of peroxidases. Cancer cells maintain elevated levels of ROS, which activate tumor-promoting pathways involving hypoxia-inducible factor (HIF), phosphoinositide 3-kinase (PI3K), nuclear factor kappa-light-chain-enhancer of activated B cells (NF-κB), and mitogen-activated protein kinase (MAPK) [45]. However, excessively high ROS levels can cause severe damage to nucleotides, proteins, cell membranes, and organelles, ultimately leading to cell death. To counteract this potentially lethal accumulation, cellular antioxidant defense mechanisms continuously modulate ROS levels, striving to maintain redox homeostasis. In Fenton and Fenton-like reactions, divalent iron reacts with hydrogen peroxide to generate hydroxyl radicals, a primary source of radical formation [12]. Mitochondria also serve as significant sources of ROS and are essential for lipid peroxidation and ferroptosis. Within the mitochondria, electron leakage from complexes I and III of the electron transport chain generates superoxide, which is subsequently converted to hydrogen peroxide by superoxide dismutase (SOD2). Light exposure can activate photosensitizing agents that generate ROS, a process known as triggered ROS. The accumulation of ROS leads to the opening of the mitochondrial permeability transition pore (mPTP) and dissipation of the mitochondrial membrane potential, resulting in a surge of ROS in a phenomenon termed ROS-induced ROS release (RIRR) [46]. The NADPH oxidase family, which includes NOX1, NOX3, and NOX4, contributes to endogenous ROS production. The ROS generated by NOX1, CYBB, and NOX4 function as intracellular second messengers that regulate various cellular processes [47–49]. To eliminate harmful ROS, intracellular antioxidant defense mechanisms, including SOD, catalase, and peroxidase, actively detoxify these oxidatively active substances. Additionally, mitochondrial enzymes, particularly SOD, neutralize superoxide radicals, generating oxygen and hydrogen peroxide. These byproducts are subsequently detoxified by glutathione peroxidase, further contributing to cellular redox balance.

Given the close association between ROS and the mechanisms of ferroptosis, studies on ROS have substantially advanced the understanding of ferroptosis in cancer biology. Mitofusin 2 (MFN2) mediates the fusion of mitochondria to increase mROS, functioning as a ferroptosis promoter. However, under ferroptotic pressure, the E3 ubiquitin ligase RBCK1 promotes the K48-linked ubiquitination and degradation of MFN2, which confers ferroptosis resistance in PDAC [50]. Additionally, CuCP molecules can be integrated into a liposomal-based nanosystem to form biocompatible and stable CuCP nanoparticles (CuCP Lipo NPs). Within the TME, these nanoparticles can be reduced to Cu⁺ by GSH, which subsequently reacts with hydrogen peroxide via a Fenton-like reaction to generate hydroxyl radicals, ultimately inducing ferroptosis in PDAC cells [51]. DIAPH3 enhances the expression of TrxR1, a component of the thioredoxin system, by increasing selenium levels. This mechanism effectively inhibits ferroptosis through the reduction of ROS concentrations in PDAC cells [52].

### System Xc^−^/GSH/GPX4 axis and PDAC

Solute carrier family 7 member 11 (SLC7A11), in conjunction with SLC3A2, forms system Xc^−^, a cystine/glutamate antiporter. This sodium-independent system exchanges intracellular glutamate for extracellular cystine at a 1:1 ratio. In the extracellular environment, cysteine is oxidized to form cystine, a dimer of two cysteine molecules joined by a disulfide bond. Cystine is then imported into the cell via system Xc^−^, where the SLC7A11 subunit plays a critical role in maintaining redox homeostasis due to its specificity for transporting cystine and glutamate and SLC3A2 acts as a molecular chaperone that stabilizes SLC7A11 at the plasma membrane. In tumor cells, elevated oxidative stress significantly increases the demand for GSH, leading to the upregulation of system Xc^−^ in many cancers. Following its uptake, cystine is rapidly reduced back to cysteine by NAD (P)H. Notably, when extracellular cysteine levels are high, cysteine can be directly imported into the cell by transporters such as the alanine-serine-cysteine (ASC) system. Cysteine, along with glutamate and glycine, synthesizes GSH, a tripeptide where cysteine acts as the rate-limiting precursor. GPX4 then uses GSH to reduce lipid hydroperoxides to lipid alcohols, thereby suppressing ferroptosis [53]. Glutathione peroxidase 4 (GPX4), a member of the GPX family, has three isoforms: nuclear, cytosolic, and mitochondrial. Among them, cytosolic GPX4 effectively disrupts the lipid peroxidation chain reaction by reducing complex hydroperoxides [54]. GPX4’s catalytic activity depends on a key triad structure consisting of selenocysteine at position 46, aspartic acid at position 81, and tryptophan at position 136 [55]. The ROS clearance mediated by GPX4 involves a two-step catalytic cycle. First, the selenocysteine residue at the active site reduces ROS, being oxidized in the process. Then, the oxidized selenocysteine is reduced back to its active form by GSH, thereby regenerating GPX4 activity. Given the pivotal role of the system Xc^−^-GSH-GPX4 axis in ferroptosis defense, inhibiting GPX4 or SLC7A11 has emerged as a promising strategy to induce ferroptosis in PDAC. Various modulators, such as LONP1 [56], SMAD4 [57], Thiostrepton (TST) [58], verteporfin [59], and tomatidine [60], regulate GPX4 expression at the transcriptional level to induce ferroptosis in PDAC cells. PIR and NFIC bind to specific promoter regions of GPX4 and act as a pair of complementary regulators of GPX4 expression. Macrophage-capping protein (MCP) suppresses the UFMylation of PIR, leading to a reduction in GPX4 transcription [61]. Additionally, N6F11, a newly identified ferroptosis inducer, binds to the RING domain of tripartite motif containing 25 (TRIM25). This interaction triggers TRIM25-mediated K48-linked ubiquitination of GPX4 and induces ferroptosis [62]. System Xc^−^ also plays a key role in the defense against ferroptosis in PDAC. The tumor suppressor protein p53 reduces SLC7A11 expression via transcriptional regulation, thereby promoting ferroptosis [63]. Similarly, miR-139-5p transcriptionally suppresses SLC7A11 to induce ferroptosis and inhibits the proliferation, invasion, and metastasis of PDAC cells [64]. Additionally, the long noncoding RNA LINC00578 binds to UBE2K, disrupting its interaction with SLC7A11 and inhibiting the ubiquitination of SLC7A11, thus promoting PDAC cell invasiveness and resistance to ferroptosis [65]. Gene-silencing nanoparticles targeting SLC7A11 have shown promise in reducing the activity of CAFs, which contribute to PDAC progression by creating a physical barrier to therapeutic agents [66]. Autophagy also influences ferroptosis by regulating SLC7A11. Under conditions where autophagy is compromised, mTORC2 phosphorylates SLC7A11 at Ser26, promoting its inactivation through its translocation from the plasma membrane to lysosomes [67, 68].

Several pharmacological compounds have been shown to induce ferroptosis in PDAC by simultaneously inhibiting SLC7A11 and GPX4. For example, WJ460 inhibits the oncoprotein myoferlin, triggering mitophagy and ROS accumulation while reducing both SLC7A11 and GPX4 expression [69]. ZZW-15, an inhibitor of nuclear protein 1 (NUPR1), downregulates SLC7A11 and GPX4 mRNA levels and induces ferroptosis in MiaPaCa-2 cells [70]. Therefore, targeting the System Xc^−^/GSH/GPX4 axis while minimizing side effects represents a major ongoing challenge and a key direction for future PDAC therapy.

### The NAD (P)H-FSP1-CoQH_2_/VKH_2_ pathway and PDAC

Coenzyme Q (CoQ), also known as ubiquinone (UQ), is a lipophilic, redox-active molecule present in all eukaryotic species. Owing to its widespread distribution in cells, CoQ acts as an electron carrier in the mitochondrial respiratory chain [71]. CoQ exists in three distinct redox states: fully reduced (CoQH₂), partially reduced (ubisemiquinone, CoQ·^−^), and fully oxidized (CoQ), and its ability to transition between these states enables its function as an electron transporter [72]. During electron transfer within the electron transport chain, premature single-electron reduction can lead to the generation of superoxide radicals. In the respiratory chain, CoQ transfers electrons from complexes I and II to complex III. The Fe‒S centers of reduced FMN transfer their electrons to CoQ in two steps: the first step forms the transient CoQ·⁻, whereas the second step reduces it to fully reduced CoQH₂. Notably, CoQ·^−^ can react with oxygen, generating superoxide radicals. In addition to its role in electron transport, CoQH₂ serves as an endogenous antioxidant that protects proteins from carbonylation, shields DNA from oxidative damage, and neutralizes lipid peroxyl radicals, safeguarding membrane lipids from peroxidation. These findings establish CoQH₂’s role as a ferroptosis suppressor, protecting cells from lipid peroxidation and oxidative stress. Vitamin K is catalyzed by UbiA prenyltransferase domain-containing protein 1 (UBlAD1) in the ER. Like CoQ in structure, vitamin K consists of a (naphtho)quinone group and an FPP-derived polyisoprenyl tail. These proteins share the same binding pocket as FSP1 [73].

The NAD (P)H-FSP1-CoQH_2_/VKH_2_ system has been identified as a parallel ferroptosis defense pathway to the SLC7A11-GSH-GPX4 axis in nonmitochondrial environments [73]. Ferroptosis suppressor protein 1 (FSP1) was initially identified as a mitochondrial pro-apoptotic protein and was formerly known as AIFM2. The myristoylation of FSP1 targets the protein to the plasma membrane, where FSP1 facilitates the NAD (P)H-dependent reduction of CoQ [5]. Another study identified vitamin K as an additional substrate catalyzed by FSP1 to its reduced form, VKH₂ [15]. The reduced CoQH₂ or VKH_2_ generated in this process functions as a reactive thiol antioxidant (RTA), preventing the propagation of lipid peroxides. As a result, FSP1 is recognized as a key glutathione-independent ferroptosis suppressor [6, 74].

In PDAC, the overexpression of cytochrome P450 2J2 (CYP2J2) leads to elevated levels of 8,9-DHET, which upregulates FSP1 mRNA and contributes to ferroptosis resistance in PDAC. Additionally, the long noncoding RNA LINC01133 forms a complex with FUS and FSP1, stabilizing FSP1 and inhibiting ferroptosis. The suppression of LINC01133 reverses ferroptosis resistance and promotes ferroptosis in PDAC cells [75]. Another regulator, TRIM21, binds to FSP1 through its PRY-SPRY domain and mediates K63 ubiquitination, facilitating FSP1 translocation to the plasma membrane. The TRIM21-FSP1 pathway protects digestive system tumor cells from ferroptosis and represents a potential therapeutic target for cancer treatment [76]. As the key driver oncogene of PDAC, KRAS has been reported to upregulate FSP1 expression to confer ferroptosis resistance and facilitate the establishment of PDAC. Consequently, the use of FSP1 inhibitors combined with ferroptosis-inducing therapies is recommended for treating KRAS-mutant cancers, including PDAC [77].

### Other pathways in ferroptosis

Tetrahydrobiopterin (BH4), a member of the pteridine family, is widely distributed among natural heterocyclic compounds. Pteridine ring systems can exist in several oxidation states, including the oxidized form dihydrobiopterin (BH2) and the reduced form BH4 [78]. The primary function of BH4 is as a cofactor for monooxygenase enzymes [78]. Additionally, BH4 is essential for various physiological processes, such as maintaining oxidative balance, protecting against nitric oxide (NO) toxicity, and mediating inflammation. Notably, GCH1-expressing cells synthesize BH4/BH2, which prevents the depletion of phospholipids containing two polyunsaturated fatty acyl tails, thereby suppressing ferroptosis [79].

The de novo synthesis of BH4 occurs through three steps. Firstly, GTP is first converted into 7,8-dihydroneopterin triphosphate (NH2-triP) by GCH1, the rate-limiting enzyme. NH2-triP is then transformed into 6-pyruvoyltetrahydrobiopterin (6-PTP) by 6-pyruvoyl tetrahydrobiopterin synthase (PTPS), and finally, 6-PTP is reduced to BH4 via three successive reactions catalyzed by sepiapterin reductase (SPR). When dihydropteridine reductase (DHPR) activity is absent or low, BH2 is generated from qBH2 and subsequently reduced back to BH4 via the salvage pathway. This recycling pathway itself is essential for regenerating BH4 after it is consumed as a cofactor by aromatic amino acid hydroxylases in the synthesis of monoamines and nitric oxide.

The overexpression of GCH1 has been shown to mitigate lipid peroxidation, positioning the GCH1-BH4 pathway as an antioxidant mechanism that inhibits ferroptosis [79]. Beyond its direct protective role, this pathway is subject to regulation by multiple factors in cancer cells. For example, the novel circRNA circLRFN5 binds to and degrades the PRRX2 protein through ubiquitin-mediated proteasomal degradation, downregulating PRRX2-driven GCH1 expression in glioblastomas (GBMs). This leads to decreased BH4 levels and consequently induces ferroptosis [80]. Methyltransferase 3 (METTL3)-mediated N6-methyladenosine (m6A) modification is another cancer-promoting mechanism that protects cells from ferroptosis by increasing PBX1 mRNA stability, which in turn increases GCH1 expression and inhibits ferroptosis through the GCH1-BH4 axis [81]. Additionally, dihydrofolate reductase (DHFR) regenerates BH4 from BH2, thus inhibiting ferroptosis. Inhibition of DHFR could induce ferroptosis effectively in cancer [82]. Supplementing BH4 can reverse DHFR inhibition-induced ferroptosis in GCH1 knockout cells [82, 83]. Despite these findings, the role of the GCH1-BH4 pathway in the ferroptosis of PDAC remains to be fully elucidated, necessitating further research.

Dihydroorotate dehydrogenase (DHODH) is a critical enzyme that converts dihydroorotate (DHO) to orotate while reducing CoQ to CoQH_2_ [84, 85]. DHODH, in parallel with GPX4, suppresses mitochondrial lipid peroxidation by reducing CoQ to CoQH_2_. DHODH and mitochondrial GPX4 complement each other [86], suggesting that DHODH inhibitors could be potential treatments for GPX4-deficient cancers [87]. However, another study indicated that DHODH inhibitors may eliminate ferroptosis resistance by inhibiting FSP1 rather than DHODH, suggesting that the role of DHODH in ferroptosis resistance may be minimal [88].

**Fig. 2:**
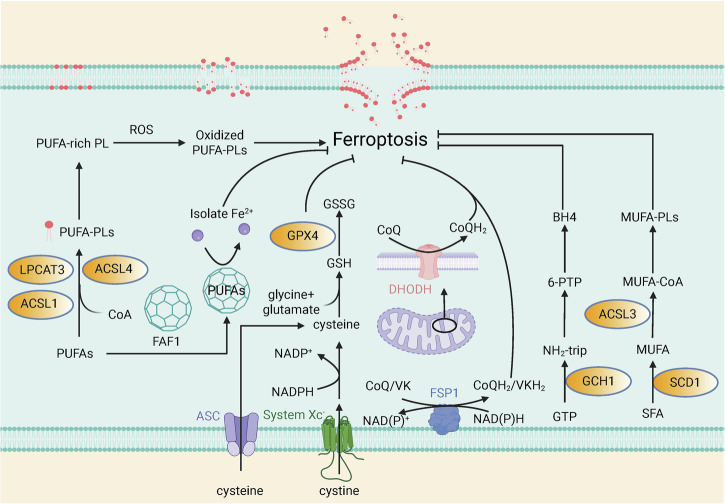
The initiating factors and defense systems of ferroptosis. PUFA-PL synthesis and accumulation of lipid peroxides promote ferroptosis. The system Xc-/GSH/GPX4, NAD (P)H-FSP1-CoQH_2_/VKH_2_, DHODH-CoQH_2_ and GCH1-BH4 systems, together with MUFA-PL synthesis, are the major defense systems against ferroptosis. PUFA polyunsaturated fatty acid, LPCAT3 lysophosphatidylcholine acyltransferase 3, ACSL4 long-chain fatty acid–CoA ligase 4, ACSL1 long-chain fatty acid–CoA ligase 1, CoA coenzyme A, FAF1 FAS-associated factor 1, PL phospholipid radical, ROS reactive oxygen species, ASC alanine-serine-cysteine, (P)LOOH (phospho)lipid hydroperoxide, (P)LOH (phospho)lipid alcohol, (P)LOO· (phospho)lipid peroxyl radicals, GSH glutathione, GSSG oxidized glutathione, NAD (P)H nicotinamide adenine dinucleotide phosphate, CoQ coenzyme Q, DHODH dihydroorotate dehydrogenase, VK vitamin K, FSP1 ferroptosis suppressor protein 1, GTP guanosine triphosphate, GCH1 GTP cyclohydrolase 1, NH_2_-triP 7,8-dihydroneopterin triphosphate, 6-PTP 6-pyruvoyltetrahydrobiopterin, BH4 tetrahydrobiopterin, SFA saturated fatty acids, SCD1 stearoyl-CoA desaturase 1. This figure was created with BioRender.com.

Beyond its pro-apoptotic role, FAS-associated factor 1 (FAF1) also suppresses ferroptosis by inhibiting PUFA peroxidation. Through its UAS domain, FAF1 binds free PUFAs and forms a protective globular structure that isolates them from pro-oxidant iron, thus preventing lipid peroxidation [89]. Exploring FAF1 function could therefore provide new avenues for triggering ferroptosis and enhancing cancer therapy (Fig. 2).

## Role of ferroptosis in PDAC development

### The tumor suppressive role of ferroptosis

Genetic alterations in PDAC often involve key oncogenes and classical cancer signaling pathways. Whole-exome sequencing of PDAC has revealed mutations and somatic copy number alterations (SCNAs) in tumor suppressor genes, including RAS, TP53, SMAD4, and BAP1. KRAS is the predominant mutated RAS gene in cancers, with the G12D mutation being the most common in PDAC [90, 91]. This mutation is associated with reduced susceptibility to conventional chemotherapy. Oncogenic KRAS drives tumorigenesis by promoting fatty acid biosynthesis [92], upregulating ACSL3 to reprogram lipid homeostasis [93], and inducing NRF2 expression to activate the antioxidant system [94]. This activation enhances GSH synthesis and promotes GPX4 activity [94–97]. Given the pivotal role of KRAS in cancer cell metabolism, targeting this oncogene could be a viable strategy to inhibit PDAC development. Additionally, ADP-ribosylation factor 6 (ARF6), a downstream effector of the KRAS/ERK signaling pathway, promotes the proliferation of PDAC cells. Silencing ARF6 increases ACSL4 and SCD1 levels, sensitizing PDAC cells to gemcitabine by inducing ferroptosis [98].

The tumor protein p53 is another key mutation in PDAC and is present in more than 50% of patients. p53 regulates lipid metabolism by controlling proteins such as NPC1L1, EPLIN, CEL, and PLTP. In addition to its role in systemic lipid metabolism, p53 inhibits lipid synthesis by suppressing NADPH production. It also decreases SLC7A11 expression by lowering H2Bub1 levels through the nuclear translocation of the deubiquitinase USP7, thereby increasing ferroptosis sensitivity [99, 100]. Paradoxically, p53 can also protect cancer cells from ferroptosis with induced GLS2 transcription and GSH/GSSG ratio, and reduced ROS [16, 101, 102]. Specific TP53 mutations can alter lipid peroxidation levels and the response to ferroptosis inducers such as erastin. Notably, R273-mutant TP53-expressing cells exhibit increased lipid peroxidation [103]. In contrast, cold-inducible RNA-binding protein (CIRBP), which is upregulated by cold exposure, binds directly to p53, inhibits its expression, and promotes ferroptosis in PDAC [104]. MMRi62 has also demonstrated anti-PDAC activity by inducing proteasomal degradation of p53 in PDAC cells [105].

Other oncogenes implicated in PDAC development, such as SMAD4 and BAP1, also play anticancer roles through ferroptosis regulation. Loss of SMAD4 occurs in more than 50% of PDAC patients [106, 107]. SMAD4 is essential for the TGF-β1-induced epithelial‒mesenchymal transition (EMT), and can downregulate GPX4 expression, consequently increasing vulnerability to ferroptosis. Gemcitabine combined with the ferroptosis inducer RSL3 is more effective in killing SMAD4-positive PDAC cells than in killing SMAD4-negative cells [57]. BRCA1-associated protein 1 (BAP1), a nuclear deubiquitinase involved in DNA damage repair, is mutated in approximately 2.9% of PDAC patients [108]. BAP1 reduces SLC7A11 expression by decreasing H2Aub occupancy on its promoter, leading to diminished cystine uptake, enhanced lipid peroxidation, and ferroptosis [109].

The Keap1‒Nrf2 pathway, a key mechanism in antioxidant defense and xenobiotic detoxification, is frequently upregulated in PDAC cells, making it a promising therapeutic target. Cytoplasmic polyadenylation element binding protein 1 (CPEB1), downregulated in PDAC, normally promotes p62 translation. The resulting decrease in p62 leads to reduced sequestration of Keap1, thereby increasing Nrf2 stability and activating transcription of anti-ferroptosis genes [110]. Additionally, GOT1, which is upregulated in exosomes from patients with PDAC, promotes the expression of its binding protein CCR2 and upregulates both Nrf2 and HO-1, contributing to ferroptosis resistance in patients with PDAC [111]. Another study revealed that HSP90α competitively binds to Keap1, stabilizing Nrf2, which results in the upregulation of GPX4 and promotes ferroptosis resistance in PDAC [112]. Moreover, the mitochondrial calcium uniporter (MCU) increases mitochondrial ROS (mROS), activating the Keap1-Nrf2 antioxidant response and upregulating SLC7A11, which enhances cystine uptake and inhibits ferroptosis in PDAC [113].

**Fig. 3:**
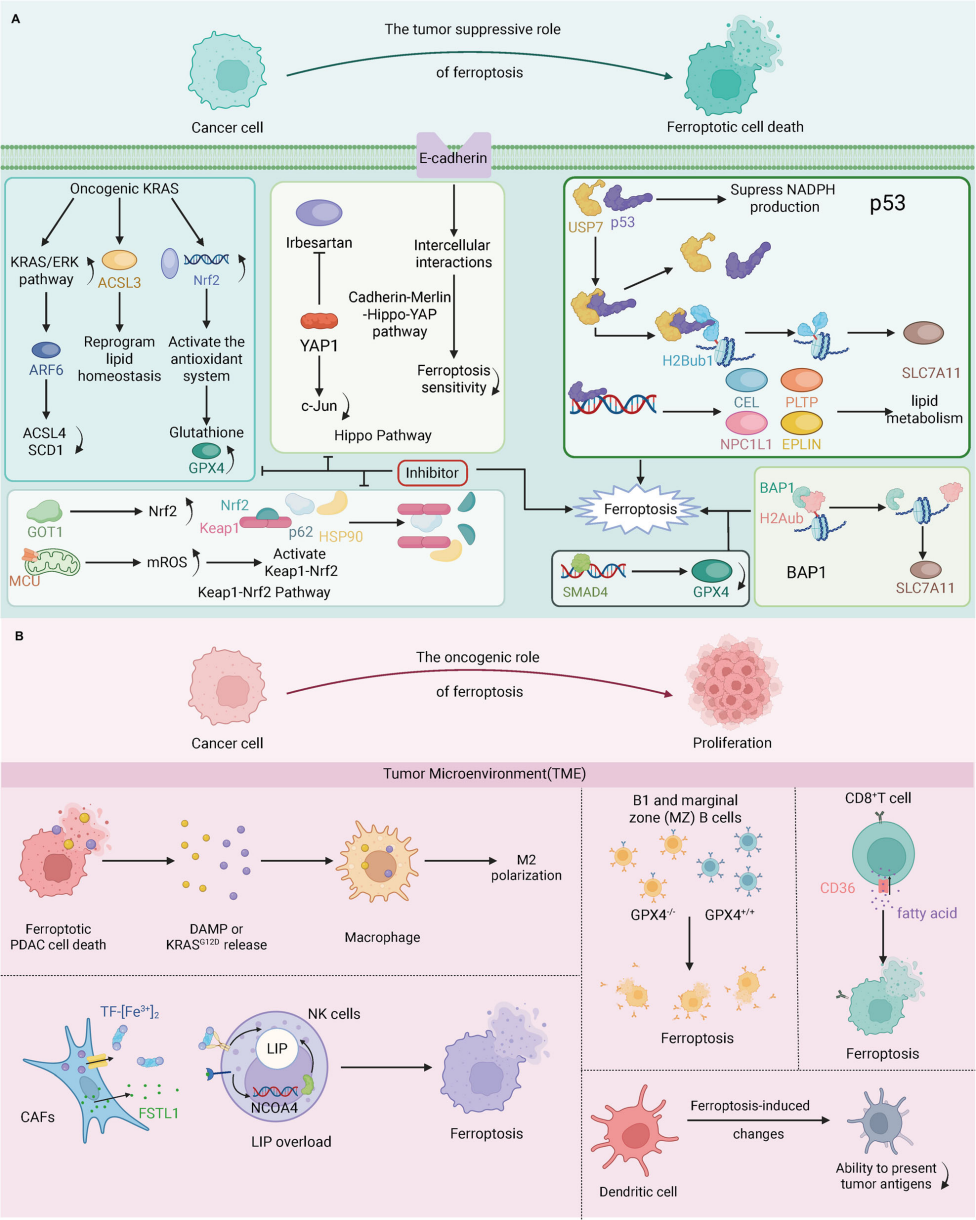
Dual roles of ferroptosis in PDAC. **A** The tumor suppressive role of ferroptosis. (1) Oncogenic KRAS reprograms lipid homeostasis by upregulating ACSL3, promotes GPX4 activity and glutathione synthesis by activating the antioxidant system and downregulating ACSL4 and SCD1. HSP90, GOT1 and MCU activate the Keap1-Nrf2 pathway. Irbesartan and intercellular interactions mediated by E-cadherin suppress ferroptosis via the Hippo pathway. Inhibitors of the KRAS, Keap1-Nrf2 and Hippo pathways trigger ferroptosis. (2) P53 suppresses NADPH production; decreases SLC7A11 expression by reducing H2Bub1; and regulates NPC1L1, EPLIN, CEL and PLTP to increase ferroptosis sensitivity. SAMD4 downregulates GPX4, and BAP1 reduces SLC7A11 expression by decreasing H2Aub occupancy to increase ferroptosis. **B** The oncogenic role of ferroptosis. DAMPs) or KRAS^G12D^ proteins released from ferroptotic cells are internalized by macrophages, promoting M2 polarization. TF-[Fe^3+^]_2_ and FSTL1 from CAFs induce ferroptosis in NK cells. Ferroptosis of B cells, CD8 + T cells, dendritic cells and other APCs promotes tumorigenesis. SMAD4 mothers against decapentaplegic homolog 4, BAP1 ubiquitin carboxyl-terminal hydrolase BAP1, H2Aub histone H2A ubiquitination, p53 cellular tumor antigen p53, USP7 ubiquitin carboxyl-terminal hydrolase 7, CEL bile salt-activated lipase, NPC1L1 NPC1-like intracellular cholesterol transporter 1, PLTP phospholipid transfer protein, EPLIN epithelial protein lost in neoplasm, ARF6 ADP-ribosylation factor 6, YAP1 transcriptional coactivator YAP1, GOT1 glutamic-oxaloacetic transaminase 1, Nrf2 nuclear factor erythroid 2-related factor 2, MCU calcium uniporter protein, mitochondrial, mROS mitochondrial reactive oxygen species, Keap1 Kelch-like ECH-associated protein 1, HSP90 heat shock protein 90 homolog, DAMP damage-associated molecular pattern, CAFs cancer-associated fibroblasts, FSTL1 Follistatin-related protein 1, NK cells natural killer cells, NCOA4 nuclear receptor coactivator 4, LIP labile iron pool, CD36 cluster of differentiation 36. This figure was created with BioRender.com.

The Hippo signaling pathway, a well-established tumor suppressor mechanism, is also a key determinant of ferroptosis [114]. Irbesartan, an angiotensin receptor blocker, inhibits the activation of the Hippo/YAP1/TAZ signaling pathway, decreasing c-Jun expression, which upregulates iron metabolism genes and suppresses ferroptosis. This contributes to resistance to gemcitabine in patients with PDAC [115]. Moreover, E-cadherin-mediated intercellular interactions downregulate TFRC and ACSL4, reducing ferroptosis sensitivity via the cadherin-Merlin-Hippo-YAP signaling pathway. Merlin knockdown reverses this decrease in ferroptosis sensitivity, suppressing tumor progression [116]. PDAC cells exhibit a complex landscape of mutations in tumor suppressor genes and dysregulation of critical signaling pathways, including Keap1-Nrf2 and Hippo. These alterations collectively contribute to ferroptosis resistance. Targeting these mutations and pathways presents a promising strategy for re-sensitizing PDAC cells to ferroptosis and inhibiting tumor progression (Fig. 3A).

### The oncogenic role of ferroptosis

While ferroptosis is primarily known for its tumor-suppressive function, it can also paradoxically promote tumor progression through its effects on immune cells and the TME. This dual nature of ferroptosis creates complex conditions that can support tumor growth, immune evasion, and metastasis. First, ferroptosis shapes the TME in ways that promote immune suppression and tumor progression. Tumor-associated macrophages (TAMs), particularly those of the M2 phenotype, are activated by ferroptosis-induced oxidative damage [117]. For example, damage-associated molecular patterns (DAMPs) or KRAS^G12D^ proteins released from ferroptotic cells can be packaged into exosomes and internalized by macrophages, transforming them into the M2-like phenotype and leading to an immunosuppressive environment [118]. Moreover, the increased presence of lipid peroxidation products and iron accumulation in the TME creates oxidative stress, further driving tumorigenesis [119].

On the other hand, ferroptosis can directly impair the antitumor immune response by negatively affecting key immune cells, including natural killer (NK) cells, B cells, cytotoxic T cells, and antigen-presenting cells (APCs), which are critical components of antitumor immune defense. In PDAC, CAFs with elevated levels of ferroportin and ferritin increase the iron pool in NK cells [120]. Moreover, CAFs also secrete FSTL1, which upregulates NCOA4, a receptor involved in ferritinophagy, thereby inducing ferroptosis in NK cells [120]. The resulting reduction in NK cell activity weakens their ability to eliminate tumor cells. GPX4 deficiency also triggers ferroptosis in B1 and marginal zone (MZ) B cells, potentially contributing to tumorigenesis [121]. In melanoma models, CD36 mediates fatty acid uptake, leading to ferroptosis in CD8 + T cells and impairing their antitumor capacity [122]. In addition, ferroptosis-induced changes in the TME can suppress the activity of APCs, including dendritic cells, hindering their ability to present tumor antigens to T cells, increasing the survival advantage of cancer cells [123]. Through modifying TME and disrupting immune cell function, ferroptosis promotes immune escape and drives tumorigenesis (Fig. 3B).

## Role of ferroptosis in PDAC diagnosis

PDAC remains one of the most challenging cancers to diagnose and treat because of its aggressive nature and typically late detection, with a 5-year survival rate of less than 5% [124, 125]. Currently, the gold standard for diagnosing PDAC involves histopathology and cytology analyses, with specimen collection methods including endoscopic ultrasonography (EUS), computed tomography (CT)-guided biopsy, ascites cytology, and exploratory biopsy during laparoscopy or open surgery [126]. Tumor biomarkers, along with CA242, carcinoembryonic antigen (CEA), CA125, microRNAs, and KRAS gene mutations, also play crucial roles in diagnosing PDAC, with commonly used markers including CA19-9 for postoperative detection, recurrence monitoring, and prognosis [127–130]. Recently, additional biomarkers, such as B7-H4 (associated with poor prognosis), circulating cell-free DNA (cfDNA), and mutation-specific circulating cell-free tumor DNA (cftDNA), have been identified as potential indicators of tumor burden and volume [131–133]. Combining tumor markers with imaging techniques such as CT and positron emission tomography (PET-CT) provides a comprehensive view of tumor morphology and molecular characteristics, enhancing early detection of PDAC [134]. However, improving early diagnosis, treatment, and prognosis remains a pressing challenge, necessitating the discovery of new molecular markers.

Recent studies have identified ferroptosis-related genes (FRGs) as potential biomarkers for diagnosing and predicting the prognosis of patients with PDAC. Serum ferritin (SF) stands out as a reliable and easily measurable marker of total body iron stores and is elevated in patients with PDAC compared with healthy controls [135]. Several ferritin-associated genes show differential expression across pancreatic tumor grades. These include key ferroptosis regulators such as ALOX15, CBS, FDFT1, RPL8, TP53, and TTC35, as well as iron homeostasis genes like MYC and FXN. The varying expression levels of these genes could potentially serve as indicators of tumor progression and severity [136]. Moreover, the hepcidin-ferroptosis axis, which regulates systemic iron metabolism [137, 138], has also been implicated in PDAC. Genes associated with this axis, including HJV, TFR2, TFR1, BMP6, and HAMP [20, 139], are significantly dysregulated in PDAC, further linking ferroptosis and iron homeostasis to tumor progression [140]. Therefore, ferroptosis-related markers, especially those involved in iron regulation, could serve as diagnostic and prognostic tools in PDAC.

The exploration of ferroptosis-related biomarkers continues to expand, with several genes and molecular pathways showing promise for improving PDAC diagnosis and prognosis. Centrosome and spindle pole-associated protein (CSPP1) has emerged as a potential diagnostic biomarker. CSPP1 promotes ferroptosis and is upregulated in various cancers, including PDAC, suggesting its utility in early detection and disease monitoring [141]. Zinc finger protein 488 (ZNF488), which promotes the transcription of stearoyl-CoA desaturase 1 (SCD1), confers resistance to ferroptosis in PDAC. The positive correlation between ZNF488 and SCD1 suggests that both genes could serve as biomarkers for diagnosing and monitoring ferroptosis resistance in PDAC [142]. CIRBP, a protein that promotes ferroptosis under cold stress, is downregulated in PDAC tissue compared with adjacent normal tissues [104]. This reduced expression highlights the potential role of CIRBP in early diagnosis and prognosis. The lncRNA A2M-AS1, a promoter of ferroptosis, is expressed at low levels in PDAC, and its downregulation is correlated with poor prognosis [143]. Similarly, the LINC02432/Hsa-miR-98-5p/HK2 axis is negatively correlated with ferroptosis, and is linked to PDAC development and prognosis. High levels of LINC02432, low levels of Hsa-miR-98-5p, and high HK2 expression are associated with a poor prognosis, underscoring their potential as biomarkers in PDAC [144]. While research on ferroptosis-related biomarkers in PDAC has rapidly expanded, it’s important to note that much of this work has focused on a limited number of genes and lacks thorough validation in clinical settings. Future studies should aim to validate these findings in larger, diverse patient cohorts and explore the integration of multiple markers for improved diagnostic and prognostic accuracy. Additionally, investigating the functional roles of these biomarkers in PDAC progression could provide insights into novel therapeutic strategies. As our understanding of ferroptosis in PDAC continues to grow, so does the potential for developing more effective diagnostic, prognostic, and therapeutic approaches for this challenging malignancy (Table 2).

**Table 2:** Advanced biomarkers targeting ferroptosis in PDAC diagnosis.

Gene	Gene product	Working mechanism	Expression in PDAC	Ref.
FTL, FTH	SF	SF reflects iron stores	↑	[135]
ALOX15	Arachidonate 15-lipoxygenase	ALOX15 catalyzes the peroxidation of PUFAs and acts as the mediator of ferroptosis	↓	[136]
CBS	Cystathionine beta-synthase	CBS catalyzes the synthesis of GSH and resists ferroptosis	↓	[136]
FDFT1	Squalene synthase	FDFT1 promotes ferroptosis through the AKT signaling pathway	↓	[136]
HAMP	Hepcidin	Hepcidin regulating intracellular iron level through modulating FPN	↑	[139]
RPL8	Large ribosomal subunit protein uL2	RPL8 is a ferroptosis driver	↑	[136]
TP53	Cellular tumor antigen p53	P53 regulates SLC7A11, SAT1 and DPP4 to promote or inhibit ferroptosis	↓	[136]
TTC35	ER membrane protein complex subunit 2	TTC35 is a mitochondrial gene that suppresses erastin-induced ferroptosis	↑	[136]
MYC	Myc proto-oncogene protein	MYC is involved in regulating iron hemostasis and positively correlated with FTL	↑	[136]
FXN	Frataxin	FXN is involved in regulating iron hemostasis	↓	[136]
HJV	Hemojuvelin	HJV is involved in the production of hepcidin, iron absorption and accumulation	↑	[139]
TFR2	Transferrin receptor protein 2	TFR2 regulates HAMP transcription and hepcidin expression by monitoring extracellular iron	↑	[139]
TFR1	Transferrin receptor protein 1	TFR1 regulates HAMP transcription and hepcidin expression by monitoring extracellular iron	↑	[139]
BMP6	Bone morphogenetic protein 6	BMP6, together with HJV, is involved in the production of hepcidin, iron absorption and accumulation	↑	[139]
CSPP1	Centrosome and spindle pole-associated protein 1	CSPP1 is involved in ferroptosis-related pathways and associated with TP53 mutation	↑	[141]
ZNF488	Zinc finger protein 488	ZNF488 promotes SCD1-mediated unsaturated fatty acid metabolism and suppresses the ferroptosis	↑	[142]
SCD1	Stearoyl-CoA desaturase	SCD1 catalyzes unsaturated fatty acid metabolism	↑	[142]
A2M-AS1	LncRNA A2M-AS1	A2M-AS1 binds to PCBP3 to promote ferroptosis	↓	[143]
LINC02432	LINC02432	LINC02432/hsa-miR-98–5p/HK2 axis is correlated with ferroptosis suppressor gene and sorafenib sensitivity	↑	[144]
MIR98	Hsa-miR-98-5p	LINC02432/hsa-miR-98–5p/HK2 axis is correlated with ferroptosis suppressor gene and sorafenib sensitivity	↓	[144]
HK2	Hexokinase-2	LINC02432/hsa-miR-98–5p/HK2 axis is correlated with ferroptosis suppressor gene and sorafenib sensitivity	↑	[144]
CIRBP	Cold-inducible RNA-binding protein	CIRBP induces ferroptosis via p53/GPX4 pathway	↓	[104]

## Role of ferroptosis in PDAC treatment

For PDAC treatment, the current standard typically involves surgery followed by adjuvant chemotherapy [145]. However, owing to the lack of effective early detection and screening methods, most patients are diagnosed at an advanced or metastatic stage, making them ineligible for surgical intervention [145]. Even among those who undergo surgery, recurrence within the first year is common. Nowadays, immunotherapy has made progress in treating various cancers, including lung cancer, breast cancer, and liver cancer. These include immune checkpoint inhibitors (ICIs) targeting PD-1/PD-L1, such as pembrolizumab and nivolumab, which have exhibited encouraging results in early clinical trials [146, 147]. However, ICIs have shown limited efficacy in treating PDAC, predominantly because of the hostile TME characterized by fibrosis, hypoxia, and immunosuppression [148]. Given the aggressive nature of PDAC and the limitations of current treatments, targeting ferroptosis has emerged as a promising therapeutic approach.

Recent advancements in the understanding of ferroptosis have highlighted its potential as a novel therapeutic strategy for treating PDAC. Ferroptosis leads to the destruction of cancer cells by triggering iron-dependent oxidative damage, indicating ferroptosis as a promising approach in PDAC therapy. Erastin, a well-known ferroptosis inducer, inhibits system Xc^−^ and blocks voltage-dependent anion channels, thereby activating p53 and inducing ferroptosis [149, 150]. However, some PDAC cells exhibit reduced sensitivity to erastin-induced ferroptosis. Combining erastin with vitamin C has been shown to increase intracellular ferrous iron levels via the AMPK/Nrf2/HMOX1 axis, resensitizing PDAC cells to ferroptosis and presenting a novel therapeutic approach [151]. WJ460 is a small compound binding to myoferlin, can reduce the expression of GPX4 and sensitizes PDAC cells to ferroptosis. The combination of WJ460 with ferroptosis inducers such as erastin and RSL3 produce synergistic effects for PDAC therapy [69]. Another compound, wogonin, has been reported to inhibit the Nrf2/GPX4 axis, leading to the accumulation of lipid peroxides and iron, thereby inducing ferroptosis in PDAC [152].

Over the past decade, gemcitabine has remained the standard first-line therapy for advanced PDAC, primarily exerting its effects by inducing DNA damage, promoting ROS accumulation, and triggering ferroptosis [153]. However, resistance to gemcitabine often develops within weeks of treatment, presenting a significant therapeutic challenge. The mechanisms underlying this resistance are complex and involve multiple enzymes and signaling pathways linked to nucleoside metabolism. Given that gemcitabine induces ferroptosis as part of its anticancer mechanism, combining ferroptosis-inducing therapies with gemcitabine has emerged as a promising strategy to overcome drug resistance. Notably, the influence of temperature within tumor tissue on ferroptosis and gemcitabine resistance in PDAC. An increase in temperature within PDAC tumor tissue drives alterations in lipid metabolism, including a decrease in PUFAs such as phosphatidylcholine plasmalogen (PC-P), attenuating lipid peroxidation and ferroptosis, and further drives gemcitabine resistance in PDAC through the p38-MAPK pathway [154]. Studies have also identified several molecular targets involved in gemcitabine resistance that modulate ferroptosis sensitivity. For example, SLC38A5 has been implicated in gemcitabine resistance by reducing lipid ROS through the regulation of GSH levels and the mTOR-SREBP1 signaling pathway. Inhibition of SLC38A5 enhances the sensitivity of gemcitabine-resistant pancreatic cells to ferroptosis [155]. Similarly, exosomes secreted by CAFs, which contain miR-3173-5p, suppress ferroptosis by inhibiting ACSL4 after gemcitabine treatment. Targeting miR-3173-5p could improve the therapeutic efficacy of gemcitabine [42]. ARF6 inhibits RSL3-induced ferroptosis by modulating ACSL4. Knocking down ARF6 sensitizes PDAC cells to ferroptosis and mitigates gemcitabine resistance. This is partly due to the upregulation of gemcitabine-related proteins such as DCK and hENT1 when ARF6 is silenced [98]. Additionally, phosphatase and tensin homolog (PTEN) plays a critical role in mediating gemcitabine resistance. Knocking down ARID3A has been shown to increase PDAC cell sensitivity to gemcitabine by reducing the expression of GPX4 in a PTEN-dependent manner [156]. The small molecule quinolinol MMRi62 has also demonstrated potential in overcoming gemcitabine resistance by promoting the proteasomal degradation of mutant p53 and the lysosomal degradation of NCOA4 and FTH1, ultimately inducing ferroptosis. MMRi62 may be particularly effective in treating PDAC patients with TP53 mutations [105]. Additionally, the inhibition of N-glycosylation, which is essential for the membrane localization and interaction of CD98hc with xCT, enhances the sensitivity of PDAC cells to ferroptosis. Treatment with tunicamycin (TM), an N-glycosylation inhibitor, in combination with gemcitabine significantly reduces the viability of PANC-1 cells, suggesting that targeting the N-glycosylation pathway holds promise for combination therapy in PDAC [157]. Collectively, these findings underscore the potential of targeting ferroptosis-related pathways to overcome gemcitabine resistance in patients with PDAC.

Furthermore, nanoparticles and liposomes have shown promise in enhancing the delivery of chemotherapy drugs to tumor sites. One innovative approach involves the use of platelet vesicles (PVs) encapsulating RSL3, which has synergistic effects through the combination of the tumor-targeting properties of PVs with the ferroptosis-inducing capability of RSL3 [158]. This system exhibits excellent biosafety, biocompatibility, and minimal organ toxicity, making it a promising therapeutic option. Another innovative approach employs PTFE nanoparticles, comprising an erastin-loaded polylactic-coglycolic acid (PLGA) core encapsulated by a metal-organic framework (MOF) shell. This shell is formed through the coordination between iron (Fe) and tannic acid. These nanoparticles exert antitumor activity through the combined effects of erastin-induced ferroptosis and the Fenton reaction, both of which contribute to increased ferroptosis [159].

Additionally, gemcitabine-loaded carbonaceous nanoparticles (MFC-GEM) have been developed to enhance the therapeutic effects of ferroptosis. The MnFe_2_O_4_ component of MFC-GEM depletes GSH, increases ROS levels, and enables pH-responsive release of gemcitabine in vivo. The accumulation of MFC-GEM also allows for simultaneous MRI monitoring in PDAC, indicating that it can serve as a contrast agent [160]. These nanoparticle-based approaches represent promising advancements in improving drug delivery and enhancing the efficacy of ferroptosis-inducing therapies in PDAC. Therapeutic approaches for PDAC related to ferroptosis are summarized in Table 3.

**Table 3:** Drugs or targets against ferroptosis in PDAC.

Compound/target	Mechanism	Effect on ferroptosis	Types of models	Research progress	Ref.
Erastin	Erastin inhibits system Xc^−^, blocks the voltage-dependent anion channel and activates p53	Induction	PANC-1, CFPAC1, MIA PaCa-2, and PANC2.03 cells	Erastin is one of the most classical ferroptosis inducers	[149, 150]
WJ460	WJ460 binds to myoferlin and reduces the expression of GPX4	Sensitization	PANC-1 cells, BxPC-3 cells	WJ460 increase the efficiency of erastin and RSL3 synergistically	[69]
Wogonin	Wogonin inhibits the Nrf2/GPX4 axis and leads to the accumulation of lipid peroxides and iron	Induction	PANC-1 and AsPC-1 cells	Wogonin targeting Nrf2 axis could reverse the drug resistance	[152]
Gemcitabine	Gemcitabine induces DNA damage, promotes ROS accumulation	Induction	PANC-1 and BXPC-3 cells	Gemcitabine has been shown to induce ferroptosis and ferroptosis evasion confers gemcitabine resistance in PDAC	[153, 154, 185–187]
SLC38A5	SLC38A5 reduces lipid ROS through the regulation of GSH levels and the mTOR-SREBP1 signaling pathway	Sensitization	PANC-1 and Capan-1 cells	Inhibition of SLC38A5 reverses gemcitabine-resistance	[155]
ACSL4	Inhibition of ACSL4 suppresses ferroptosis	Inhibition	PANC-1 and MIA PaCa-2 cells	Targeting miR-3173-5p inhibits ACSL4 to reverse gemcitabine-resistance; Knockdown of ARF6 modulates ACSL4 to mitigate gemcitabine-resistance	[42]
ARID3A	Knockdown of ARID3A reduces GPX4	Sensitization	MIA PaCa-2, Capan-1 and PANC-1 cells; cell derived xenograft models, patient specimens from patients with primary PDAC	Knockdown of ARID3A enhance gemcitabine sensitivity	[156]
MMRi62	MMRi62 promotes the proteasomal degradation of mutant p53 and the lysosomal degradation of NCOA4 and FTH1	Induction	PANC-1 and BxPc3 cells	MMRi62 shows efficiency in PDAC with TP53 mutation and gemcitabine-resistance	[105]
N-glycosylation	Inhibition of N-glycosylation enhances ferroptosis sensitivity	Sensitization	PANC-1, BxPC-3, AsPC-1, and MIA PaCa-2 cells; Patient specimens from patients with primary PDAC	N-glycosylation inhibitor with gemcitabine holds promise for PDAC	[157]
PVs encapsulating RSL3	PVs targets tumor and RSL3 induces ferroptosis	Induction	PANC-1, PANC-2 and MIA PaCa-2 cells	PV and RSL3 show synergistic effect	[158]
PTFE	PTFE induces both erastin-induced ferroptosis and Fenton reaction	Induction	KPC1199 mouse-derived primary cancer cells; cell-derived xenograft models	PTFE demonstrates anti-tumor effect with ferroptosis-inducing and tumor penetration capabilities	[159]
MFC-GEM	MFC-GEM increases ROS level	Induction	BALB/c mice; PANC02 and hTERT-HPNE cells	MFC-GEM not only enhances ferroptosis but also serves as contrast agent in PDAC simultaneous MRI monitoring	[160]

*SLC38A5* solute carrier family 38 Member 5, *ACSL4* long-chain-fatty-acid-CoA ligase 4, *ARF6* ADP-ribosylation factor 6, *ARID3A* Arid family member 3A, *MFC-GEM* gemcitabine-loaded carbonaceous nanoparticles, *PVs* platelet vesicle.

## Limitations and future perspectives

PDAC persists as a therapeutic challenge due to its aggressive biology, propensity for late-stage detection, and high frequency of drug resistance. Ferroptosis represents a promising therapeutic avenue, as its core mechanisms align with the metabolic dependencies of PDAC. Realizing this potential, however, requires addressing significant limitations and charting new research directions. A primary obstacle is the incomplete understanding of the molecular mechanisms governing ferroptosis in this context. While four major defense systems, including the GPX4-GSH axis, FSP1-CoQ_10_ pathway, DHODH-CoQ_10_ system, and GCH1-BH4 pathway, are known to regulate redox homeostasis and lipid peroxidation, their inhibition elicits heterogeneous responses across PDAC models. This variation points to the involvement of tumor-specific regulatory mechanisms that remain elusive. Growing evidence underscores a complex crosstalk between ferroptosis and other key cellular processes, including metabolic reprogramming, autophagy, and immune regulation, all of which collectively shape ferroptosis susceptibility. For instance, the contribution of lipid metabolism, particularly PUFA biosynthesis, to ferroptosis sensitivity in PDAC is not fully defined. Similarly, interactions with iron homeostasis pathways, such as ferritinophagy and the hepcidin-ferroptosis axis, may unveil novel therapeutic targets. Deciphering these underlying networks is crucial for identifying the unique ferroptotic vulnerabilities of PDAC cells.

The adverse effects of ferroptosis in cancer therapy must be carefully considered alongside its therapeutic potential. Ferroptosis-based therapies offer significant advantages, including personalized treatment tailored to genetic profiles, potential to overcome drug resistance, and synergy with other therapeutic modalities [161]. However, the potential side effects cannot be overlooked. Notably, hematopoietic stem cells (HSCs) exhibit high sensitivity to ferroptosis inducers like erastin and RSL3, among others [162]. This sensitivity can result in bone marrow suppression, increasing the risk of hematological complications. Second, many ferroptosis inducers are metabolized in the liver and excreted through the kidneys [163, 164]. This metabolic pathway may inadvertently induce ferroptotic damage in hepatic and renal tissues, potentially compromising organ function. Third, studies have revealed that the induction of ferroptosis in mice can lead to early-onset cachexia and reduced survival rates, highlighting the systemic impact of ferroptosis on noncancerous tissues [165]. Addressing these challenges necessitates a deeper investigation into the distinct ferroptotic vulnerabilities of malignant cells. Identifying and targeting tumor-specific ferroptosis pathways while protecting normal tissues will help mitigate these adverse effects. This strategy could lead to the development of highly selective ferroptosis-inducing therapies, optimizing tumor cell elimination while preserving healthy tissues, thereby enhancing both the safety and efficacy of ferroptosis-based treatments for PDAC.

The interaction between ferroptosis and the TME remains poorly defined but is increasingly recognized as a critical area of research. Immune cells and their associated cytokines in the TME exhibit a dual capacity to modulate ferroptosis in cancer cells. For example, components such as transforming growth factor beta 1 (TGF-β1) and interferon gamma (IFN-γ), which are secreted by CD8^+^ T cells and neutrophils, have been shown to enhance ferroptosis in cancer cells [166, 167]. However, additional studies suggest that the release of TGF-β via anoctamin 1-mediated pathways facilitates the recruitment of CAFs and impairs the antitumor immunity mediated by CD8^+^ T cells, demonstrating the dual role of TGF-β in the TME [168]. Second, ferroptotic cancer cells orchestrate intricate responses in the antitumor immune landscape within the TME. They release immunostimulatory signals that can activate dendritic cells, M1-polarized macrophages, and T cells, thereby promoting an antitumor immune response [169]. Conversely, specific phospholipid peroxidation products generated during ferroptosis can impair dendritic cell maturation, whereas factors such as CXCL10 and HMGB1 released by ferroptotic cancer cells promote the infiltration of immunosuppressive polymorphonuclear myeloid-derived suppressor cells (PMN-MDSCs). These PMN-MDSCs secrete PGE1, which inhibits the activity of CD8^+^ T cells, thereby weakening antitumor immunity [167]. Third, ferroptosis inducers, while targeting tumor cells, may inadvertently impact immune cells in the TME, potentially compromising the immune response and attenuating overall antitumor efficacy. More specific targets should be identified between tumor cells and surrounding immune cells. For example, N6F11 has been reported to selectively induce ferroptosis without affecting immune cells by binding to TRIM25, which is expressed predominantly in tumor cells rather than immune cells [62]. Furthermore, the combination of N6F11 and TRIM25 enhances GPX4 protein degradation, leading to ferroptosis specifically in tumor cells [62]. Taken together, elucidating the complex interplay between ferroptosis and the TME is paramount, as the bidirectional effects of immune cells and ferroptotic cancer cells within this microenvironment can significantly modulate antitumor immunity, ultimately shaping the efficacy of PDAC therapies.

The integration of ferroptosis-related biomarkers into clinical practice could significantly enhance PDAC diagnosis and monitoring, pending validation through larger, more diverse clinical studies. Building on these biomarkers, we propose a precision medicine strategy for PDAC. For instance, while oncogenic KRAS mutations increase cellular oxidative stress and sensitize cells to ferroptosis, they concurrently upregulate FSP1 as a key resistance mechanism [77]. This rationale supports a patient-selection hypothesis wherein KRAS-mutant tumors with high FSP1 expression are uniquely vulnerable to the combination of an FSP1 inhibitor and a ferroptosis inducer. To test this, we propose a minimal feasible, biomarker-enriched, single-arm trial for this patient subset, with co-primary endpoints of safety and objective response rate (ORR). Given the potential impact of ferroptosis on the tumor microenvironment, the trial protocol would mandate rigorous monitoring for myelosuppression and immune-related adverse events. Likewise, clinical trials are essential to optimize dosage regimens, administration routes, and safety profiles of ferroptosis-inducing agents, thereby maximizing their therapeutic potential. Another critical area that requires attention is the potential of ferroptosis in overcoming gemcitabine resistance, which is a major challenge in PDAC treatment. Gemcitabine has been shown to induce PDAC cell death partially through ferroptosis [154]. Integrating ferroptosis-inducing approaches with gemcitabine therapy may offer a novel strategy to bypass conventional resistance mechanisms. Elucidating the complex relationship between gemcitabine resistance and ferroptosis is crucial for devising potent combination therapies that enhance therapeutic outcomes in PDAC. As research continues to advance, the combination of ferroptosis with existing therapies, such as chemotherapy, immunotherapy and targeted treatments, holds great potential for improving the prognosis and survival rates of PDAC patients. Notwithstanding the promise of ferroptosis in preclinical models, its clinical translation faces significant hurdles, particularly in drug delivery and on-target/off-tumor toxicity. Therefore, we explicitly state that larger clinical studies are imperative to rigorously establish the safety and efficacy of these approaches in humans. The insights gained from such studies will be critical for navigating these barriers and developing viable, evidence-based therapeutic strategies. Overcoming current limitations through dedicated research efforts will be crucial to unlocking the full therapeutic potential of ferroptosis in PDAC treatment.

## Conclusions

PDAC remains a formidable clinical challenge, characterized by a highly aggressive phenotype, a profoundly immunosuppressive TME, and notorious resistance to conventional and emerging immunotherapies. This review has synthesized the growing body of evidence positioning ferroptosis, an iron-dependent form of regulated cell death driven by lipid peroxidation, as a compelling therapeutic avenue for this recalcitrant malignancy. The core mechanisms underpinning ferroptosis—dysregulated iron metabolism, peroxidation of PUFA-containing phospholipids, and compromised antioxidant defense systems—converge on the unique metabolic vulnerabilities of PDAC cells. Notably, the oncogenic drivers prevalent in PDAC, such as mutant KRAS and loss of TP53 or SMAD4, intricately modulate key ferroptotic pathways, including the System Xc⁻/GSH/GPX4 axis, the FSP1-CoQH₂ system, and lipid metabolism networks, thereby dictating cellular susceptibility to ferroptotic stress.

The role of ferroptosis in PDAC is context-dependent and exhibits a stark duality. On one hand, targeted induction of ferroptosis presents a powerful strategy to eliminate tumor cells, particularly those resistant to apoptosis, and can synergize with standard chemotherapeutics like gemcitabine to overcome treatment resistance. On the other hand, ferroptosis within the TME can paradoxically foster tumor progression by impairing the antitumor functions of immune cells such as natural killer (NK) cells and cytotoxic T lymphocytes, and by promoting an immunosuppressive landscape. This duality underscores the critical need for spatially and temporally controlled induction strategies. The emergence of FRGs and proteins as potential diagnostic and prognostic biomarkers further highlights the clinical translatability of this pathway, offering hope for improved early detection and patient stratification.

Looking forward, the clinical application of ferroptosis-based therapies in PDAC hinges on overcoming several pivotal challenges. First, a deeper mechanistic understanding of the crosstalk between ferroptosis and PDAC-specific signaling networks is essential to identify novel, highly selective targets. Second, the development of sophisticated delivery systems, such as nanoparticles and biomimetic vesicles, is crucial to enhance the bioavailability and tumor-specific targeting of ferroptosis inducers while minimizing off-target toxicity to normal tissues. Finally, the intricate and often opposing effects of ferroptosis on antitumor immunity demand careful consideration when designing combination regimens with immunotherapy. Strategic integration of ferroptosis inducers with agents that remodel the TME towards an immunostimulatory state could unlock synergistic antitumor efficacy. Concerted efforts to address these aspects will be instrumental in harnessing the full potential of ferroptosis modulation, ultimately paving the way for transformative therapeutic strategies against PDAC.
